# Oxeiptosis – potential in cancer treatment?

**DOI:** 10.1017/erm.2026.10045

**Published:** 2026-03-27

**Authors:** Mateusz Kciuk, Katarzyna Wanke, Żaneta Kałuzińska-Kołat, Renata Kontek

**Affiliations:** 1 Department of Molecular Biotechnology and Genetics, Faculty of Biology and Environmental Protection, University of Lodz, Lodz, Poland; 2 Doctoral School of Exact and Natural Sciences, University of Lodz, Lodz, Poland; 3 Department of Functional Genomics, Faculty of Medicine, Medical University of Lodz, Lodz, Poland

**Keywords:** cell death, chemotherapy, oxeiptosis, oxidative stress, reactive oxygen species (ROS)

## Abstract

Oxeiptosis is a reactive oxygen species (ROS)-dependent form of programmed cell death that plays a key role in cellular homeostasis and holds promise as a cancer therapy. This review explores its molecular mechanisms, emphasizing the KEAP1–PGAM5–AIFM1 signalling pathway and its reliance on ROS accumulation. Compared to other cell death pathways, oxeiptosis offers a distinct approach, especially for targeting cancer cells resistant to conventional therapies. The review evaluates emerging inducers, both synthetic and natural, that selectively trigger oxeiptosis in cancer cells. It also examines the potential synergy between oxeiptosis and ROS-generating chemotherapies, particularly in the oxidative tumour microenvironment. However, challenges remain, including identifying tumour-specific inducers, overcoming cancer cell resistance to oxidative stress and reducing off-target effects. The review concludes by highlighting the need for targeted delivery strategies and rigorous preclinical studies to translate oxeiptosis into effective cancer treatments. Overall, it underscores oxeiptosis as a promising avenue to address drug resistance and improve therapeutic outcomes in oncology.

## Introduction

Historically, cancer treatment has heavily relied on classical chemotherapy as a cornerstone of therapeutic intervention. The primary objective of chemotherapy is to impede the rapid proliferation of malignant cells, which characteristically undergo more frequent and uncontrolled divisions than normal cells. Chemotherapeutic agents function by interfering with key cellular processes essential for cancer cell growth, ultimately leading to apoptosis or other forms of cell death. However, due to the nonspecific nature of traditional chemotherapeutic drugs, which affect all rapidly dividing cells, healthy cells (particularly those in the bone marrow, gastrointestinal tract and hair follicles) are also susceptible to damage. This lack of selectivity results in a range of adverse effects, including myelosuppression, gastrointestinal disturbances and alopecia, which can significantly impact patients’ quality of life and limit the therapeutic dose (Refs. 1–3). Furthermore, resistance to chemotherapy is a significant challenge, greatly hindering its effectiveness. Chemotherapy resistance occurs when cancer cells adapt to evade the cytotoxic effects of chemotherapeutic agents designed to inhibit their growth or induce cell death. This resistance can be categorized as either intrinsic (present before treatment) or acquired (developed during the treatment). The mechanisms underlying chemotherapy resistance are complex and multifactorial, involving genetic, molecular and cellular adaptations. These include: (1) acquisition of mutations that render chemotherapeutic drugs less effective, (2) increase in the expression of efflux pumps, which actively transport chemotherapeutic drugs out of the cells, (3) and upregulation of enzymes that detoxify or inactivate chemotherapeutic drugs, (4) enhanced DNA repair capabilities in cancer cells that counteract the DNA-damaging effects of chemotherapy, and lastly (5) changes in the pathways that regulate cell death can lead to chemotherapy resistance (Refs. 4–6).

Apoptosis is a tightly regulated process that allows the body to eliminate damaged or abnormal cells, which is essential for maintaining tissue homeostasis. This form of cell death ensures the efficient removal of cellular debris, preventing the release of harmful substances into surrounding tissues and avoiding inflammatory responses. In contrast, unregulated (such as necrosis) and programmed cell deaths (necroptosis (Ref. 7) and pyroptosis (Ref. 8)) can provoke inflammation and cause further tissue damage. On the contrary, they also may have a significant role in immune responses against tumours (Refs. 9, 10). In line with this evidence, our previous works extensively covered apoptosis, necroptosis or pyroptosis (Ref. 11) and metal-dependent cell death pathways, including ferroptosis and cuproptosis (Ref. 12) induction as the paths for the elimination of cancer cells. Nevertheless, other understudied cell death pathways can also be activated by chemotherapeutic drugs in certain conditions. Among these, oxeiptosis has emerged as a particularly intriguing form of cellular demise.

Oxeiptosis is a regulated form of cell death specifically triggered by oxidative stress, distinguished by its non-inflammatory nature that plays a crucial role in maintaining cellular homeostasis under conditions of prolonged exposure to high levels of detrimental reactive oxygen species (ROS). This non-inflammatory characteristic is particularly relevant in cancer therapy, where inflammation can amplify tissue damage and complicate clinical outcomes. The process of oxeiptosis is mediated through the Kelch-like ECH-associated protein 1 (KEAP1), phosphoglycerate mutase family member 5 (PGAM5) and mitochondrial apoptosis-inducing factor 1 (AIFM1) pathway, which senses oxidative stress and initiates downstream signalling to execute cell death. Leveraging the KEAP1 pathway to induce oxeiptosis presents a potential strategy in cancer treatment. Such an approach could improve therapeutic efficacy by selectively targeting cancer cells under oxidative stress while minimizing inflammation-related side effects, offering a dual advantage in cancer therapy by enhancing tumour cell clearance and reducing treatment-associated toxicity (Ref. 13).

Although oxeiptosis was first described only recently, accumulating evidence suggests that it represents a distinct and biologically relevant oxidative stress–induced cell death pathway that is not fully captured by existing classifications of regulated cell death. To date, oxeiptosis has largely been discussed in isolation or mentioned only briefly within broader redox or cell death reviews, with limited integration into cancer biology and therapy-oriented frameworks. The novelty of the present review lies in (i) systematically positioning oxeiptosis within the broader landscape of ROS-driven regulated cell death pathways, (ii) critically synthesizing emerging mechanistic insights linking KEAP1–PGAM5–AIFM1 signalling to cancer-associated redox dysregulation, and (iii) comprehensively evaluating experimental evidence suggesting that oxeiptosis may contribute to the anticancer effects of diverse therapeutic modalities, including chemotherapeutic agents, natural compounds, nanomedicine-based platforms and combination regimens. Importantly, this review also extends beyond oncology by highlighting recent proof-of-concept studies demonstrating spatially controlled oxeiptosis induction in non-malignant hyperproliferative diseases, underscoring its broader biological relevance.

## Mechanisms of oxeiptosis

ROS are generated naturally as by-products of cellular metabolism, primarily within mitochondria, peroxisomes and other organelles. Cancer cells typically exhibit elevated ROS levels due to several factors, including altered mitochondrial function. In cancer cells, dysfunctional mitochondria can leak electrons from the electron transport chain (ETC), which results in the formation of superoxide radicals and subsequently other ROS, such as hydrogen peroxide (H₂O₂) and hydroxyl radicals. Additionally, the high metabolic demands of rapidly proliferating cancer cells drive increased cellular respiration and energy production, which in turn enhances ROS production as a metabolic by-product. Tumours often contain hypoxic regions, areas with low oxygen availability, that further contribute to elevated ROS levels. Hypoxia impairs mitochondrial efficiency and activates ROS-generating enzymes, further amplifying ROS production.

A key aspect of cancer cell biology is the frequent impairment of DNA repair pathways, which leads to genomic instability. Elevated ROS levels, combined with deficient DNA repair, promote mutations and genetic diversity within the tumour, potentially supporting cancer cell adaptation, survival and proliferation in a hostile microenvironment. While high ROS levels are cytotoxic, cancer cells often develop mechanisms to balance ROS production with antioxidant defences, enabling them to exploit ROS for signalling and adaptive advantages without succumbing to oxidative damage (Ref. 14).

Although ROS contribute to cancer progression by promoting cellular survival, proliferation and genomic instability, excessive ROS accumulation can also induce cell death via several mechanisms. One of the primary forms of ROS-induced cell death is apoptosis. ROS can initiate apoptosis by activating key signalling pathways, including the tumour suppressor pathway mediated by cellular tumour antigen p53 (TP53). This pathway responds to oxidative stress by promoting the expression of pro-apoptotic genes. Additionally, ROS can directly oxidize proteins critical to the regulation of apoptosis, such as members of the BCL-2 family, thereby altering their function and promoting cell death. Understanding the dual role of ROS in both supporting tumour progression and inducing cell death highlights their complex role in cancer biology and suggests that modulating ROS levels could be a potential therapeutic strategy to selectively target cancer cells (Ref. 15). ROS can also induce necroptosis, a regulated form of necrosis, through activation of receptor-interacting protein kinases (RIPKs) and subsequent formation of the necrosome (Ref. 16) and ferroptosis, a regulated form of cell death characterized by iron-dependent lipid peroxidation. In ferroptosis, ROS promote the peroxidation of polyunsaturated fatty acids (PUFAs) within cellular membranes, leading to membrane damage and eventual cell death. This process is heavily reliant on iron, which catalyses the formation of lipid peroxides through the Fenton reaction, thereby amplifying ROS production and promoting ferroptotic cell death. Another emerging ROS-induced cell death pathway is cuproptosis, a form of cell death driven by copper-dependent ROS production. Cuproptosis involves the accumulation of copper within cells, which promotes ROS generation and disrupts mitochondrial function, leading to cell death (Ref. 12).

Recent research has identified an additional form of ROS-induced cell death, termed ‘*oxeiptosis*’, which may significantly contribute to the cytotoxic effects of ROS. Initial reports on oxeiptosis were published in 2018, arising from studies utilizing an *in vivo* ozone-exposure model in which mice exposed to ozone exhibited increased ROS levels, specifically within airway epithelial cells. Interestingly, neither genetic deletion of key genes nor pharmacological inhibition of essential proteins associated with conventional cell death pathways, such as apoptosis, ferroptosis, pyroptosis or necroptosis, was sufficient to reduce ozone-induced tissue damage, suggesting the presence of a distinct cell death mechanism. Further *in vitro* studies using H₂O₂ as a ROS inducer reinforced these observations. When canonical cell death pathways were inhibited or key pathway components were genetically depleted, a range of cell types continued to undergo ROS-induced cell death, indicating an alternative pathway at play. Critically, these experiments demonstrated that KEAP1 functionality is essential for H₂O₂-induced cell death, establishing it as a central regulator in the oxeiptotic response. This discovery of KEAP1-dependent oxeiptosis highlighted a novel dimension of oxidative stress responses and suggested potential therapeutic implications for selectively inducing cell death in ROS-rich environments, such as tumours (Ref. 13).

KEAP1 is a crucial regulatory protein in the cellular defence against oxidative stress, primarily through its role in modulating the nuclear factor erythroid 2-related factor 2 (NRF2) pathway. This pathway governs the expression of a range of antioxidant and cytoprotective genes critical for maintaining redox balance and protecting cells from ROS-induced damage. Under basal conditions, KEAP1 binds NRF2 in the cytoplasm, facilitating its ubiquitination and subsequent proteasomal degradation, thus preventing NRF2 from activating antioxidant responses in the absence of oxidative stress. Upon exposure to oxidative stress, ROS modify specific cysteine residues in KEAP1. These modifications alter KEAP1’s conformation, reducing its affinity for NRF2 and impairing its ability to target NRF2 for ubiquitination. As a result, NRF2 escapes proteasomal degradation, accumulates in the cytoplasm and translocates into the nucleus. Once in the nucleus, NRF2 binds to antioxidant response elements (AREs) located in the promoter regions of target genes, initiating the transcription of various genes involved in ROS detoxification and redox homeostasis. Key NRF2 target genes include those encoding antioxidant enzymes and redox-regulating proteins such as glutathione peroxidase (GPX), glutathione synthetase (GSS), peroxiredoxin (PRX) and thioredoxin (TRX). Together, these proteins mitigate oxidative damage by neutralizing ROS, recycling antioxidants and maintaining cellular redox balance (Refs. 17–19).

**Figure 1.**
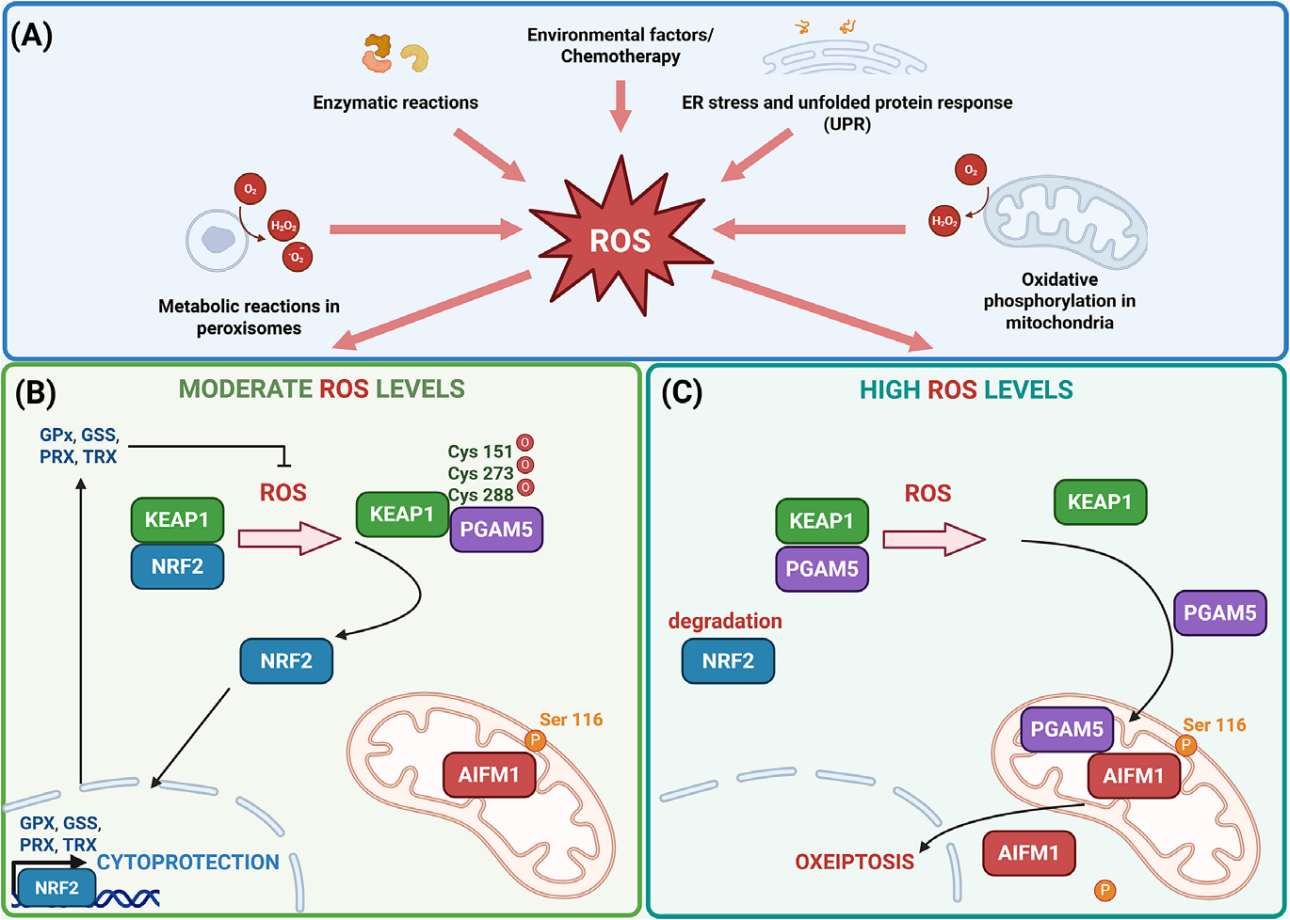
(A) Reactive oxygen species (ROS) are generated as natural by-products of cellular metabolism, primarily within mitochondria, peroxisomes and other organelles, as a result of endoplasmic reticulum (ER) stress and unfolded protein response (UPR) or as a consequence of environmental triggers including chemotherapeutic drugs. Cancer cells typically exhibit elevated ROS levels due to several factors, including altered mitochondrial function. In cancer cells, dysfunctional mitochondria can leak electrons from the electron transport chain (ETC), which results in the formation of superoxide radicals and subsequently other ROS, such as hydrogen peroxide and hydroxyl radicals. Additionally, the high metabolic demands of rapidly proliferating cancer cells drive increased cellular respiration and energy production, which in turn enhances ROS production as a metabolic by-product. (B) Exposure of cells to moderate levels of ROS induces conformational changes in Kelch-like ECH-associated protein 1 (KEAP1) by oxidizing specific cysteine residues, namely cysteine at positions 151, 273 and 288. These oxidative modifications result in the dissociation of the KEAP1-NRF2 complex, allowing nuclear factor erythroid 2–related factor 2 (NRF2) to translocate into the nucleus. Once in the nucleus, NRF2 activates the transcription of cytoprotective genes, for example glutathione peroxidase (GPX), glutathione synthetase (GSS), peroxiredoxin (PRX) and thioredoxin (TRX) that help mitigate oxidative damage by promoting the elimination of ROS. (C) However, when ROS levels are excessively high within the cell, KEAP1 undergoes further modifications, leading to the release of phosphoglycerate mutase family member 5 (PGAM5) from its complex with KEAP1. PGAM5 then interacts with apoptosis-inducing factor mitochondria-associated 1 (AIFM1), dephosphorylating it at Ser116. This modification triggers the activation of AIFM1 and contributes to the initiation of oxeiptosis, a form of regulated cell death (Ref. 22). Created in BioRender. Kciuk, M. (2025) https://BioRender.com/yc77nyv.

Prolonged or elevated oxidative stress can overwhelm the cellular antioxidant defence system, resulting in inadequate production of cytoprotective proteins and potentially leading to cellular damage or death. Under conditions of excessive intracellular ROS, the interaction between KEAP1 and PGAM5 is disrupted, triggering conformational changes in KEAP1. These changes release PGAM5 from the KEAP1-PGAM5 complex, enabling PGAM5 to engage with downstream signalling pathways that may contribute to cell death under severe oxidative stress. This dynamic interaction is a critical component of a broader regulatory network that senses oxidative stress and adjusts cellular responses based on ROS levels, underscoring KEAP1’s dual role in promoting cytoprotection and mediating cell death (Ref. 13). PGAM5 interacts with and dephosphorylates serine 116 (Ser116) of AIFM1, a key regulator of mitochondrial-mediated cell death. This dephosphorylation event triggers the activation of AIFM1, leading to its translocation to the nucleus, where it induces chromatin condensation and large-scale DNA fragmentation, ultimately contributing to oxeiptosis (Refs. 13, 20). Additionally, OTU deubiquitinase 1 (OTUD1) has been identified as a potential regulator of KEAP1 in the process of oxeiptosis. OTUD1 interacts with KEAP1 through its N-terminal region, which contains intrinsically disordered segments with specific ETGE motifs. These motifs play a crucial role in mediating the binding between OTUD1 and KEAP1, leading to the deubiquitination of KEAP1. This interaction may influence KEAP1’s stability and function, thereby modulating the cellular response to oxidative stress and contributing to the regulation of oxeiptosis (Ref. 21) (Figure 1).

Mutations in *KEAP1* have been linked to the progression of various cancers, including lung (Ref. 23) and prostate cancer (Ref. 24). Additionally, the expression of the *KEAP1* gene is frequently suppressed in several cancer types, including breast (Ref. 25), colorectal (Ref. 26), lung and prostate cancer (Ref. 27), often due to hypermethylation of its promoter region. This suppression impairs the function of the oxeiptosis pathway, potentially promoting the survival of transformed cells and providing resistance to cytotoxic anticancer therapies.

## Oxeiptosis in the context of other cell death pathways

ROS serve as a critical nexus in the initiation of various forms of regulated cell death. Chemotherapeutic agents often escalate ROS production to levels that exceed those already present in cancer cells, thereby triggering cell death. Two primary mechanisms contribute to the increased cellular ROS production during chemotherapy: (1) the generation of ROS by dysfunctional mitochondria, which are commonly impaired in cancer cells, and (2) the suppression or inhibition of the cellular antioxidant defence systems, which are crucial for maintaining redox balance and mitigating oxidative stress (Ref. 28).

ROS play a pivotal role in regulating apoptosis, primarily through the modulation of mitochondrial integrity and the activation of key pro-apoptotic signalling pathways. ROS can initiate apoptosis by inducing DNA damage, which activates and stabilizes the tumour suppressor protein TP53, a master regulator of cell death (Ref. 29). Upon activation, TP53 promotes the expression of pro-apoptotic proteins such as BAX and p53 upregulated modulator of apoptosis (PUMA), leading to mitochondrial outer membrane permeabilization (MOMP). This event triggers the release of cytochrome c from the mitochondria, initiating the caspase cascade and ultimately resulting in cell death. In addition to its indirect effects via TP53, ROS can directly modulate the activity of BCL-2 family proteins, including BAX and Bcl-2 homologous antagonist/killer (BAK1), which regulate MOMP (Ref. 30). Mitochondria, as the primary intracellular source of ROS, contribute to ROS accumulation due to electron leakage in the ETC. This leakage causes damage to mitochondrial DNA (mtDNA), disrupting the transcription of essential mitochondrial proteins and impairing ATP synthesis, thus creating a positive feedback loop of increased ROS production. ROS also facilitate mitochondrial permeability transition pore (MPTP) opening, further promoting mitochondria-dependent apoptosis (Ref. 31). Moreover, ROS can enhance apoptosis through endoplasmic reticulum (ER) stress by promoting the accumulation of misfolded proteins, which triggers the unfolded protein response (UPR). Under prolonged stress conditions, this can lead to apoptosis. Beyond their mitochondrial and ER effects, ROS activate mitogen-activated protein kinases (MAPK) pathways, including stress-activated protein kinase (JNK) and p38 MAPK, which promote apoptosis by phosphorylating BCL-2 family proteins and modulating transcription factors involved in cell death (Ref. 32).

ROS play a critical role in regulating necroptosis. Under certain conditions, ROS-induced oxidative stress can activate RIPKs, particularly RIPK1 and RIPK3, which facilitate the formation of the necrosome complex. This complex is essential for the phosphorylation of mixed lineage kinase domain-like protein (MLKL), leading to its translocation to the plasma membrane and its subsequent rupture. ROS can enhance necroptosis by directly oxidizing RIPK1 and RIPK3, as well as by modulating the cellular redox state, which is crucial for necroptotic signalling. Specifically, mitochondrial ROS production can oxidize key cysteine residues in RIPK1, promoting its autophosphorylation and subsequent activation of RIPK3 (Ref. 33). Furthermore, RIPK3 can amplify ROS generation by enhancing mitochondrial metabolism or activating NADPH oxidase (NOX) pathways, thereby contributing to necroptosis in various cancer cell types (Ref. 34). The morphological features of necroptosis, such as cell swelling, chromatin condensation and plasma membrane rupture, lead to the release of intracellular contents that trigger inflammatory responses in the surrounding tissue. Notably, antioxidant treatments that reduce ROS levels have been shown to inhibit tumour necrosis factor (TNF)-induced necroptosis, highlighting that the balance of ROS generation is a key determinant in necroptotic cell fate. Contrarily, NRF2 mitigates necroptosis by inducing the expression of antioxidant genes, thereby neutralizing excessive ROS and protecting against tissue damage (Ref. 35).

Ferroptosis is a regulated form of cell death characterized by iron-dependent lipid peroxidation, with ROS playing a central role in its initiation and propagation. The process begins with the Fenton reaction, where ROS, primarily generated from mitochondrial ETC activity, react with ferrous ions (Fe^2+^) to produce highly reactive hydroxyl radicals. These radicals initiate lipid peroxidation by extracting hydrogen atoms from PUFAs, resulting in the formation of PUFA radicals and subsequent PUFA peroxy radicals. As lipid peroxidation progresses, lipid hydroperoxides are generated, which compromise cell membrane integrity and ultimately lead to cell swelling, membrane rupture and ferroptotic cell death (Refs. 12, 36). Key protective mechanisms against ferroptosis include the antioxidant enzyme GPX4 (Refs. 37, 38) and the lipid repair enzyme ferroptosis suppressor protein 1 (FSP1) (Refs. 39, 40). GPX4 uses reduced glutathione (GSH) to detoxify lipid peroxides, converting them into less harmful alcohols, while GSH synthesis depends on the activity of gamma-glutamylcysteine synthetase (γ-GCS) and cystine transport mediated by the xCT antiporter. Cellular iron homeostasis also plays a critical role in ferroptosis; excessive iron accumulation enhances ROS production through the Fenton reaction, amplifying lipid peroxidation. Enzymes involved in lipid metabolism, such as long-chain acyl-CoA synthetase family 4 (ACSL4) and lysophosphatidylcholine acyltransferase 3 (LPCAT3), are also essential for incorporating PUFAs into cellular membranes, thereby facilitating the vulnerability of cells to ferroptotic stimuli (Ref. 41).

Cuproptosis is a form of regulated cell death induced by copper ions and ROS. Copper ions can catalyse the Fenton reaction, generating hydroxyl radicals from H₂O₂, which leads to oxidative stress and cellular damage. Excessive copper-induced ROS production can overwhelm cellular antioxidant defences, ultimately triggering cell death. The molecular mechanisms underlying cuproptosis involve oxidative damage to cellular macromolecules, including proteins, lipids and DNA, which disrupt cellular homeostasis and lead to cell death. Cuproptosis has been implicated in the pathogenesis of neurodegenerative diseases and cancer, where dysregulated copper homeostasis and increased oxidative stress contribute to disease progression and tissue damage (Refs. 42, 43).

Pyroptosis is a distinct form of regulated cell death characterized by the activation of inflammasomes and inflammation-associated caspases, with ROS playing a crucial role in its initiation and progression. In response to environmental stress or cellular damage, inflammasomes such as NOD-, LRR- and pyrin domain-containing protein 3 (NLRP3) and absent in melanoma 2 (AIM2) assemble, recruiting adapter proteins that facilitate the activation of pro-inflammatory caspases, particularly caspase-1. Activated caspase-1 cleaves gasdermin D (GSDMD), generating its N-terminal fragment, which forms pores in the cell membrane, leading to membrane rupture and the release of pro-inflammatory cytokines such as interleukins (IL-1β and IL-18). ROS act as upstream signalling molecules in this process by enhancing the expression of NLRP3, pro-caspase-1 and pro-IL-1β, thereby promoting pyroptosis (Refs. 8, 10). Additionally, iron-induced ROS production has been implicated in the activation of caspase-3 through mitochondrial pathways, further contributing to pyroptotic cell death (Ref. 44). Lipid ROS have also been shown to enhance GSDMD cleavage through caspase-1 activation, highlighting the critical role of mitochondrial ROS in driving NLRP3-dependent pyroptosis, particularly in macrophages (Ref. 45). This topic was recently reviewed by An et al. (Ref. 46). The dual role of pyroptosis in cancer, where it can promote inflammation that supports tumour growth, yet also potentially limit tumour progression, illustrates its complex implications in both health and disease. Dysregulation of pyroptosis can result in chronic inflammation, which is associated with various inflammatory conditions, underscoring the importance of further research into the molecular mechanisms of ROS-mediated pyroptosis and its role in disease pathogenesis (Refs. 47, 48).

Beyond ROS themselves, lipid peroxidation products act as critical downstream effectors that translate oxidative stress into specific cell death programs. Among these, 4-hydroxynonenal (4-HNE), a highly reactive α,β-unsaturated aldehyde generated during the oxidation of PUFAs, has emerged as a central mediator linking ROS accumulation to regulated cell death pathways. Lipid peroxidation is a self-propagating chain reaction initiated by ROS, leading to the formation of electrophilic aldehydes that persist longer than free radicals and diffuse across cellular compartments, thereby extending the spatial and temporal impact of oxidative stress. Unlike short-lived ROS, 4-HNE remains covalently bound to proteins, nucleic acids and phospholipids through Michael addition reactions, altering their structure and function even in the absence of ongoing lipid peroxidation (Refs. 49, 50).

4-HNE is increasingly recognized as a second messenger of oxidative stress, exerting pleiotropic biological effects that are highly concentration-, cell type–, and context-dependent. At low to moderate concentrations, 4-HNE participates in adaptive stress responses by modulating antioxidant capacity (Ref. 51), cell proliferation (Refs. 52, 53) and differentiation (Refs. 54–56). It can activate transcriptional programs through redox-sensitive pathways, including NRF2 (Refs. 51, 57) and activator protein-1 (AP-1) (Refs. 58, 59), thereby inducing detoxifying enzymes such as glutathione S-transferases (GSTs) (Refs. 60, 61) and aldehyde dehydrogenases (ALDHs) (Refs. 62–64). However, when produced at high levels or inadequately detoxified, 4-HNE becomes a potent cytotoxic mediator that amplifies oxidative damage and promotes regulated cell death (Refs. 65, 66).

Mechanistically, 4-HNE contributes to mitochondria-dependent apoptosis by modifying key components of the ETC, further exacerbating mitochondrial dysfunction and ROS production. Covalent adduction of 4-HNE to mitochondrial proteins disrupts oxidative phosphorylation, reduces ATP synthesis and promotes mitochondrial membrane depolarization, thereby facilitating cytochrome c release and caspase activation (Refs. 67–69).

Beyond apoptosis, emerging evidence implicates 4-HNE in additional regulated cell death modalities, including necroptosis (Ref. 70), pyroptosis (Ref. 71), autophagy-associated cell death (Ref. 72), ferroptosis (Ref. 73), cuproptosis and oxeiptosis as recently reviewed (Ref. 74). Collectively, these findings position 4-HNE as a central integrator of ROS signalling and lipid peroxidation–driven cell death, rather than a mere by-product of oxidative damage. Its ability to persist, diffuse and covalently modify cellular macromolecules enables it to orchestrate multiple death pathways simultaneously or sequentially, depending on cellular context. In cancer, where redox balance is already precarious, dysregulated 4-HNE metabolism may tip the balance between adaptive survival and cell death. Understanding the dual signalling and cytotoxic roles of 4-HNE may therefore open new therapeutic opportunities to selectively exploit oxidative vulnerabilities in cancer cells by steering lipid peroxidation towards lethal outcomes while sparing normal tissues (Ref. 74).

**Figure 2.**
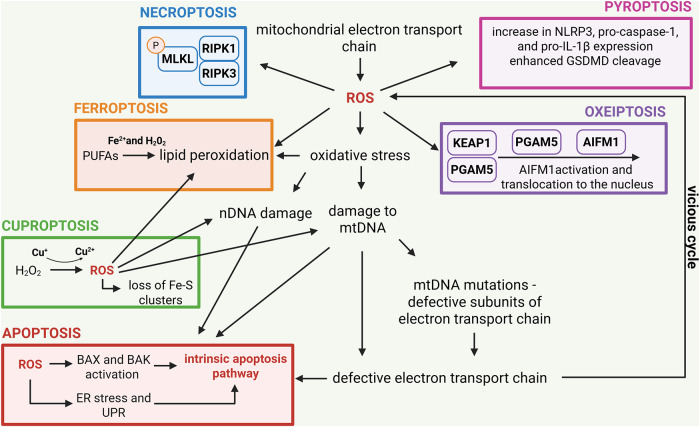
Reactive oxygen species (ROS) in the context of different types of cell death. The mitochondrial electron transport chain (ETC), ROS, oxidative stress and mitochondrial DNA (mtDNA) mutations are intricately linked in a feedback loop often described as the vicious cycle of mitochondrial dysfunction. The ETC, located in the inner mitochondrial membrane, is responsible for ATP production through oxidative phosphorylation. During this process, electron leakage from ETC complexes can lead to the formation of ROS, such as superoxide anions and hydrogen peroxide. While ROS are normal by-products of mitochondrial respiration, excessive production under pathological conditions, or ETC dysfunction causes oxidative stress, overwhelming cellular antioxidant defences. Oxidative stress is particularly detrimental to mtDNA, which resides near the ETC and lacks the protective histones and robust mechanisms of nuclear DNA (nDNA) repair. As a result, mtDNA is highly susceptible to oxidative damage, leading to mutations that impair the coding of critical ETC subunits. Dysfunctional ETC subunits exacerbate electron leakage and ROS production, creating a self-perpetuating cycle of damage. This vicious cycle promotes progressive mitochondrial dysfunction, marked by decreased ATP production, increased ROS and further mtDNA damage. Over time, this cascade contributes to different forms of cell death. The most studied is apoptosis (shown in red) in which ROS activate the intrinsic pathway as a consequence of DNA damage, pro-apoptotic BAX and BAK activation and ER stress, which can trigger UPR. Necroptosis (shown in blue) can also be triggered by ROS as a consequence of receptor-interacting protein kinases (RIPK1/3) activation and subsequent phosphorylation of mixed lineage kinase domain-like protein (MLKL) responsible for the cellular membrane pores formation. Ferroptosis (shown in orange) is marked by iron-dependent lipid peroxidation, with ROS being pivotal in its onset and progression. The process initiates with the Fenton reaction, in which ROS, predominantly produced by ETC activity, interact with ferrous ions (Fe2+) to generate highly reactive hydroxyl radicals. These radicals commence lipid peroxidation by removing hydrogen atoms from PUFAs, leading to the generation of PUFA radicals and ensuing PUFA peroxy radicals. Lipid peroxidation generates lipid hydroperoxides, which undermine cell membrane integrity, resulting in cell enlargement, membrane rupture and ferroptotic cell death. Similarly, excessive copper abundance in cells can lead to the Fenton reaction, where the loss of Fe-S clusters in proteins leads to cuproptosis (shown in green). ROS can also upregulate the expression of NLRP3, pro-caspase 1 and IL-1β expression and boost GSDMD cleavage, leading to pyroptotic cell death (shown in pink). Finally, excessive ROS production triggers KEAP1 modification, leading to the release of PGAM5 from its complex with KEAP1. PGAM5 then interacts with apoptosis-inducing factor mitochondria-associated 1 (AIFM1), dephosphorylating it at Ser116. This modification triggers the activation of AIFM1 and contributes to the initiation of oxeiptosis (shown in purple). Created in BioRender. Kciuk, M. (2025) https://BioRender.com/r8qgh1d.

Cell death pathways, including apoptosis, pyroptosis, necroptosis, ferroptosis, cuproptosis and oxeiptosis, share both common features and distinct mechanisms that govern their initiation and execution. A defining characteristic of all these pathways is their highly regulated nature and reliance on ROS, where specific molecular mediators ensure precise control over the elimination of damaged or dysfunctional cells (Figure 2).

Oxeiptosis is mechanistically distinct from other ROS-dependent cell death modalities in that it is initiated by excessive, non-mitochondrial oxidative stress and operates independently of caspase activation, MOMP, iron-dependent lipid peroxidation or inflammasome signalling. Instead, oxeiptosis is characterized by the KEAP1–PGAM5–AIFM1 signalling axis, which functions as a dedicated oxidative stress–sensing pathway. Under conditions of overwhelming ROS accumulation, KEAP1 acts as a redox sensor and releases PGAM5, leading to AIFM1 dephosphorylation and its translocation to the nucleus, where it induces large-scale DNA fragmentation and chromatin condensation without classical apoptotic features. Importantly, the specific induction of oxeiptosis appears to depend on both the intensity and the nature of oxidative stress, as well as the functional status of other cell death pathways. In contrast to apoptosis, oxeiptosis does not require TP53 activation or caspase signalling; unlike ferroptosis, it is not driven by iron-dependent lipid peroxidation or GPX4 inactivation; and unlike necroptosis or pyroptosis, it does not rely on RIPK1/RIPK3/MLKL or inflammasome–gasdermin pathways. Therefore, oxeiptosis is most likely engaged when ROS levels exceed the buffering capacity of canonical antioxidant systems but fail to efficiently trigger mitochondria-centred or lipid peroxidation–driven death programs.

## Investigated oxeiptosis inducers

While the in-depth exploration of specific inducers of oxeiptosis remains an emerging area of research, several factors and conditions relevant to cancer therapy are known to promote oxidative stress, which may, in turn, trigger oxeiptosis. For instance, in 2020, Dabaghi et al. investigated the therapeutic potential of functionalized chitosan-coated magnetic nanoparticles (CS-MNPs) loaded with 5-fluorouracil (5FU) and magnetic hyperthermia in human colon xenograft tumours. The study demonstrated that the combination of thermo-chemotherapeutic treatment (5FU-CS-MNPs and magnetic hyperthermia) resulted in a significant reduction in tumour volume and a marked decrease in tumour cell proliferation compared to either therapy used individually. The cytotoxic activity of the 5FU-CS-MNPs complex was confirmed in studies conducted on HT-29 tumour xenograft models. The thermo-chemotherapeutic treatment notably increased the expression of H2AX and phospho-H2AX (γH2AX), markers of DNA double-strand breaks and replication stress, relative to either treatment alone or the untreated control group. However, levels of poly(ADP-ribose) polymerase (PARP) and its cleaved form (c-PARP), markers of apoptosis initiation, remained unchanged after the combined treatment. Interestingly, the expression of TP53, a key protein involved in stress-induced survival responses, was elevated. In contrast, the expression of heat-shock proteins (HSP70 and HSP90), which are involved in protein stabilization, either remained unaffected or decreased compared to individual treatments. Furthermore, the levels of nuclear factor kappa-light-chain-enhancer of activated B cells (NF-κB), a marker of tumour cell proliferation, survival, and therapy resistance, were slightly reduced following the combined treatment. Despite these alterations, the expression of caspase-8, cleaved caspase-8, and caspase-3 was not significantly affected by the combined or individual therapies. When comparing the 5FU-MNP treatment to either monotherapy, the combined approach resulted in substantial tumour cell death, leading to significant and prolonged suppression of tumour development and a reduction in tumour volume, with some tumours being completely eradicated.

The authors speculated that the overproduction of ROS induced by hyperthermia, 5FU therapy, and the increased expression of H2AX may contribute to the cytotoxic effects of the treatment, possibly through the induction of oxeiptosis. Notably, no significant changes were observed in apoptotic machinery, as evidenced by unaltered caspase activity. However, the study did not investigate oxidative stress markers or other pathways associated with apoptosis or necroptosis, aside from RIPK3 phosphorylation, nor did it examine the impact of treatment on KEAP1, PGAM5 and AIFM1 pathway (Ref. 75).

In another study, Wang et al. investigated the anticancer potential of auriculasin, a naturally occurring flavonoid compound classified as a chalcone. Previous research has shown that auriculasin exerts its therapeutic effects through its pro-oxidative properties, as well as its ability to induce apoptosis and ferroptosis. These effects are evidenced by increased ROS production, mitochondrial shrinkage and the intracellular accumulation of Fe^2+^ and malondialdehyde (MDA). The study found that auriculasin upregulated the expression of KEAP1 and AIFM1, while notably decreasing the phosphorylation level of AIFM1. This regulation of KEAP1 and AIFM1 by auriculasin was effectively inhibited by the antioxidant N-acetylcysteine (NAC), suggesting that auriculasin induces oxeiptosis through ROS generation. The addition of apoptosis inhibitor (Z-VAD-FMK), ferroptosis inhibitor (FER-1), or silencing of KEAP1, PGAM5 and AIFM1 individually resulted in a significant reduction in the inhibitory effects of auriculasin on cell viability. These findings indicate that auriculasin compromises cell viability by triggering apoptosis, ferroptosis and oxeiptosis. Moreover, auriculasin demonstrated inhibitory effects on the invasion and clonogenic abilities of colorectal cancer cells (Ref. 76).

Alloimperatorin is a naturally occurring coumarin compound found in various plant species, particularly those used in traditional medicine including *Angelica dahurica.* It was suggested that this compound can induce apoptosis (Ref. 77) and autophagy (Ref. 78) in cervical cancer cells. In 2020, Zhang et al. investigated the oxeiptosis-inducing properties of alloimperatorin. The study demonstrated that alloimperatorin exhibited concentration- and time-dependent inhibition of breast cancer cell viability. Moreover, the cytotoxic effects of alloimperatorin were significantly reduced by apoptosis and ferroptosis inhibitors. Alloimperatorin effectively triggered programmed cell death in breast cancer cells, leading to increased activity of caspase-3, caspase-8, caspase-9 and PARP. Additionally, alloimperatorin caused noticeable reductions in mitochondrial size, facilitated the accumulation of Fe^2+^, ROS and MDA, and significantly decreased the expression of solute carrier family 7, subfamily A, member 11 (SLC7A11) and GPX4 at both mRNA and protein levels. These findings strongly suggest that alloimperatorin induces ferroptosis. Furthermore, alloimperatorin notably enhanced the expression of KEAP1, while not affecting the expression of PGAM5 and AIFM1. However, it significantly decreased the phosphorylation level of AIFM1. Silencing of KEAP1, PGAM5 and AIFM1 expression reduced the anticancer activity of alloimperatorin, suggesting the suppression of oxeiptosis. These findings collectively suggest that alloimperatorin triggers apoptosis, ferroptosis and oxeiptosis in cancer cells (Ref. 79).

Sanguinarine (SNG) is a potent bioactive alkaloid with a broad spectrum of biological activities, including antimicrobial, anti-inflammatory and anticancer effects (Ref. 80). Pallichankandy et al. discovered that knockdown of *KEAP1*, *PGAM5* and *AIFM1* significantly diminished SNG-induced cell death, emphasizing the role of oxeiptosis in SNG-mediated cytotoxicity. In addition, the introduction of H_2_O_2_ increased the susceptibility of colorectal cancer cells to SNG-induced oxeiptosis. Conversely, the application of ROS scavengers to eliminate intracellular ROS reversed the cell death process. Ultimately, the *in vivo* study proved that SNG efficiently inhibits tumour growth in the HT-29 xenograft mice model by inducing oxeiptosis (Ref. 81).

Similar to SNG, alantolactone (ALT) is a naturally occurring sesquiterpene lactone found in the roots of *Inula helenium*, commonly known as elecampane. ALT is recognized for its diverse pharmacological properties and has been traditionally used in herbal medicine to treat various ailments. Recent studies have shown that ALT suppresses the growth of ovarian cancer cells by significantly reducing the levels of NRF2, GSH and GPX, while simultaneously elevating the expression of PGAM5 and KEAP1. Furthermore, ALT induced the dephosphorylation of AIFM1, suggesting the initiation of oxeiptosis in treated cells (Ref. 82).

Phenethyl isothiocyanate (PEITC) is a naturally occurring compound classified as an isothiocyanate. It is derived from glucosinolates, sulphur-containing compounds found in cruciferous vegetables such as broccoli, watercress, cabbage and Brussels sprouts. Known for its distinctive pungent flavour, PEITC has garnered significant interest due to its potential health benefits and biological activities. In contrast, dasatinib is a targeted cancer therapy drug belonging to the class of tyrosine kinase inhibitors (TKIs), primarily used in the treatment of certain types of leukaemia. Strusi et al. investigated the potential synergistic effects between PEITC and dasatinib in the treatment of hepatocellular carcinoma (HCC). The combination of PEITC and dasatinib demonstrated a synergistic anticancer effect, both *in vitro* and *in vivo*, characterized by the accumulation of DNA damage, oxidative stress, mitotic catastrophe and oxeiptosis (Ref. 83).

In 2023, Lu et al. developed triphenylphosphine (TPP)-modified lipid nanoparticles encapsulating indocyanine green (ICG) as a photosensitizer and paclitaxel (PTX). Given that the majority of mitochondria are transported via microtubules in the cytoplasm, targeted delivery to mitochondria can enhance the therapeutic efficacy of PTX. The incorporation of PTX also improves the effectiveness of phototherapy, resulting in a combined anti-tumour effect that surpasses the sum of the individual treatments. As anticipated, the mitochondria-targeting nanomedicine demonstrated superior distribution to mitochondria and increased cytotoxicity against cancer cells *in vitro.* Moreover, M-ICG-PTX nanoparticles exhibited enhanced tumour growth inhibition by inducing both apoptosis and the oxeiptosis pathway, effectively suppressing primary tumour growth and metastasis. Overall, M-ICG-PTX nanoparticles represent a promising nanoplatform for achieving significant therapeutic outcomes by integrating chemotherapy with photothermal therapy (PTT) (Ref. 84).

In 2025, Cheng et al. identified magnolol as a novel inducer of oxeiptosis in lung cancer, providing mechanistic insight into how excessive oxidative stress can selectively engage this regulated cell death pathway rather than classical apoptosis or ferroptosis. Using both xenograft mouse models and *in vitro* lung cancer systems, the authors demonstrated that magnolol markedly increases intracellular ROS levels while simultaneously suppressing antioxidant defence pathways, thereby overwhelming cellular redox homeostasis. This oxidative imbalance disrupted functional crosstalk between mitochondria and peroxisomes, leading to profound mitochondrial dysfunction, impaired peroxisomal metabolism and the accumulation of damaged organelles. Importantly, magnolol-induced cell death occurred independently of caspase activation and lipid peroxidation, supporting the activation of oxeiptosis rather than apoptosis or ferroptosis. Mechanistically, magnolol promoted mitocytosis and triggered the KEAP1–PGAM5–AIFM1 axis, culminating in large-scale DNA fragmentation and chromatin condensation. A distinctive feature of this study was the demonstration that damaged mitochondria and peroxisomes were transferred between cancer cells via tunnelling nanotubes, thereby propagating oxidative stress and reinforcing oxeiptotic signalling in recipient cells. This intercellular amplification of oxidative damage led to sustained ROS accumulation and further activation of oxeiptosis, ultimately suppressing tumour growth *in vivo* (Ref. 85).

Emerging evidence suggests that oxeiptosis may also be harnessed in non-malignant hyperproliferative diseases through spatially controlled induction of oxidative stress. In this context, a recent study introduced a sonosensitizing nanoplatform based on copper–manganese-doped mesoporous silica nanoparticles (Cu–Mn@SiO₂) for ultrasound-triggered sonodynamic therapy (SDT) in benign prostatic hyperplasia (BPH). Unlike conventional pharmacological or surgical approaches, this strategy aimed to achieve ultra-minimally invasive, localized tissue modulation while minimizing off-target effects. Mechanistically, Cu–Mn@SiO₂-mediated SDT significantly enhanced intracellular ROS production in both epithelial and stromal BPH cell models, leading to activation of the oxeiptosis pathway, as evidenced by PGAM5 cleavage, mitochondrial membrane depolarization and mitochondrial structural damage. Importantly, these effects were induced in a highly controlled manner through focused ultrasound activation, underscoring the feasibility of spatially restricted oxeiptosis induction. *In vivo*, localized SDT treatment in a testosterone-induced BPH rat model resulted in marked prostate volume reduction, improved urinary function and restoration of glandular architecture, without detectable systemic toxicity or damage to surrounding tissues (Ref. 86). Studies on reported oxeiptosis inducers are summarized in Table 1.

**Table 1.** Summary of studies exploring oxeiptosis inducers

Compound/strategy	Cancer type(s) studied	Preclinical models	Key findings	Compound evaluated in clinical trials	Compound evaluated in clinical trials for cancer treatment	References (PMIDs)
Chitosan-coated magnetic nanoparticles + 5-fluorouracil + magnetic hyperthermia	Colorectal cancer	*In vitro*: HT29 human CRC cells; *in vivo*: HT29 heterotopic xenograft in mice	Combined thermo-chemotherapy reduced tumour volume, proliferation, vascularity, ROS-dependent cell death, possibly oxeiptosis	No	No	32916798
Auriculasin	Colorectal cancer	*In vitro*: CRC cell lines	Induced ROS-dependent apoptosis, ferroptosis and oxeiptosis via KEAP1/AIFM1 signalling	No	No	34872005
Alloimperatorin	Breast cancer	*In vitro*: breast cancer cell lines	Induced apoptosis, ferroptosis and oxeiptosis via KEAP1/PGAM5/AIFM1 pathway; reduced invasion	No	No	35263194
Sanguinarine (SNG)	Colorectal cancer	*In vitro*: CRC cells; *in vivo*: HT–29 xenograft mouse model	ROS (H₂O₂)–dependent activation of KEAP1-PGAM5-AIFM1 axis inducing oxeiptosis	Yes	No	36914635
Alantolactone (ALT)	Ovarian cancer	*In vitro*: SKOV3 cells	ROS-mediated oxeiptosis via downregulation of NRF2 and activation of KEAP1/PGAM5/AIFM1	No	No	37712005
Phenethyl isothiocyanate (PEITC) + dasatinib	Hepatocellular carcinoma	*In vitro*: 2D and 3D spheroids; *in vivo*: syngeneic murine HCC model	Synergistic ROS production inducing DNA damage, mitotic catastrophe and oxeiptosis	Yes	Yes	37860118 37175527 26951845
Magnolol	Lung cancer	*In vitro*: lung cancer cell lines; xenograft mouse models	Induces excessive ROS production and suppresses antioxidant signalling; disrupts mitochondrial–peroxisomal crosstalk, leading to mitochondrial dysfunction and mitocytosis; activates oxeiptosis via redox imbalance and organelle damage; promotes intercellular transfer of damaged mitochondria and peroxisomes via tunnelling nanotubes, amplifying oxidative stress and oxeiptotic signalling; suppresses tumour growth *in vivo*	No	No	40347844
Mitochondria-targeted lipid nanoparticles (ICG + paclitaxel)	Solid tumours (general, including metastasis models)	*In vitro*: cancer cell lines; *in vivo*: tumour-bearing mouse models	Enhanced mitochondrial targeting; apoptosis and oxeiptosis; strong inhibition of tumour growth and metastasis	No	No	37295777
Cu–Mn@SiO₂ sonosensitizing nanoparticles + ultrasound-induced SDT	Not cancer (benign prostatic hyperplasia)	*In vitro*: BPH–1 epithelial cells, WPMY–1 stromal cells; *in vivo*: testosterone-induced BPH rat model	SDT markedly increased intracellular ROS, inducing oxeiptosis characterized by PGAM5 cleavage, mitochondrial membrane depolarization and mitochondrial damage. Localized SDT led to ~60% reduction in prostate weight, improved urinary function, reduced fibrosis, and restored glandular morphology, with no detectable systemic toxicity.	No	No	40911431

*Abbreviations:* AIFM1: apoptosis-inducing factor mitochondria-associated 1; ALT: alantolactone; BPH: benign prostatic hyperplasia; CRC: colorectal cancer; HCC: hepatocellular carcinoma; ICG: indocyanine green; KEAP1: Kelch-like ECH-associated protein 1; NRF2: nuclear factor erythroid 2–related factor 2; PGAM5: phosphoglycerate mutase family member 5; PEITC: phenethyl isothiocyanate; ROS: reactive oxygen species; SDT: sonodynamic therapy; SNG: sanguinarine.

## Could oxeiptosis contribute to the anticancer effects of chemotherapeutic drugs?

While research on oxeiptosis is still emerging, most chemotherapeutic drugs induce oxidative stress (Ref. 14), which could potentially trigger oxeiptosis. The direct connection between specific chemotherapeutic drugs and oxeiptosis has not been extensively characterized in the literature. However, chemotherapeutic agents that generate ROS and induce oxidative stress may promote this form of cell death. Drugs like vinca alkaloids, taxanes, 5-fluorouracil, platinum-based compounds and anthracyclines can lead to ROS generation. This is often due to the impairment of mitochondrial function, which in turn contributes to oxidative stress and potentially activates oxeiptosis (Ref. 14). Here, we provide several examples of such chemotherapeutic drugs with details on their mechanism of action associated with oxidative stress:

1. Topoisomerase inhibitors, for example, doxorubicin (DOX): DOX is an anthracycline antibiotic commonly used in cancer therapy. It exerts its therapeutic effects by intercalating between DNA base pairs, thereby inhibiting topoisomerase II, an enzyme essential for DNA replication. This disruption of DNA and RNA synthesis ultimately results in cell death. DOX also interacts with cardiolipin, a phospholipid located on the inner mitochondrial membrane, triggering the generation of ROS. Elevated ROS levels significantly impact mitochondrial structure and function, which in turn promotes apoptosis in the affected cells (Refs. 87–89). Other mechanisms of DOX-induced cytotoxicity include ferroptosis, pyroptosis and autophagy. DOX treatment increases the labile iron pool within the cell and disrupts iron homeostasis by inactivating iron regulatory proteins (IRP1 and IRP2). The altered binding of inactive IRPs to iron-response elements in the promoters of various genes leads to changes in iron metabolism and protein expression. Additionally, DOX has been shown to inhibit GPX4, both in the cytosol and mitochondria, promoting lipid peroxidation. Furthermore, DOX can modulate NRF2 activity, further contributing to the induction of ferroptosis. In line with this, new formulations of DOX, including liposomes (Ref. 90) and nanoparticles (Ref. 91), were indicated as effective ferroptosis-inducing agents (Refs. 90–92). In contrast, Zhang et al. observed morphological alterations characteristic of pyroptosis, such as cellular enlargement and membrane disruption, following the treatment of MDA-MB-231 and T47D cells with DOX. GSDME was identified as a key protein in the initiation of pyroptosis triggered by DOX. Activation of caspase-3 was also noted in this process, suggesting its involvement in both pyroptosis and apoptosis. Moreover, DOX administration led to a significant accumulation of ROS within the cells (Ref. 93). DOX was also found to induce pyroptosis in melanoma cells with high intrinsic GSDME expression. In contrast, MCF-7 breast cancer cells, which have low deafness autosomal dominant 5 (DFNA5) expression, did not undergo pyroptosis under the same DOX treatment. Additionally, DOX treatment activated autophagy in the melanoma cells. Inhibition of autophagy using Beclin1 siRNA or chloroquine (20 μmol/L) significantly increased cell death through pyroptosis, thereby enhancing the sensitivity of melanoma cells to DOX. DOX treatment also activated eukaryotic elongation factor 2 kinase (eEF-2 K) in melanoma cells, and silencing of eEF-2 K reduced autophagy, subsequently triggering DOX-induced pyroptotic cell death. Consequently, eEF-2 K was identified as a key regulator in the balance between pyroptosis and autophagy in DOX-treated melanoma cells (Ref. 94).
2. Platinum compounds, for example, cisplatin: cisplatin is a potent and widely used chemotherapeutic agent with a broad spectrum of activity against various cancers. Cisplatin enters cancer cells and forms platinum-DNA adducts by binding to the DNA, primarily at the N7 position of purine bases. This binding leads to the creation of intra- and inter-strand cross links within the DNA, disrupting its double helix structure. These cross links inhibit DNA replication and transcription. In response to DNA damage, the cell activates its DNA damage response pathways. If the damage is extensive and irreparable, the cell triggers apoptosis or cell cycle arrest, primarily at the G2/M phase (Refs. 95, 96). Cisplatin can also cause mitochondrial damage, leading to increased production of ROS (Ref. 97). Furthermore, cisplatin can activate NOX, an enzyme complex that produces superoxide radicals from oxygen and depletes intracellular antioxidants such as GSH or inhibit the activity of antioxidant enzymes like superoxide dismutase (SOD), catalase and GPX4 reducing the cell’s ability to neutralize ROS (Ref. 98). The increased ROS levels induced by cisplatin can lead to lipid peroxidation, damage to cell membranes and disruption of cellular functions, ultimately triggering ferroptosis. Cisplatin disrupts cellular iron homeostasis, leading to the accumulation of free iron, which catalyses ROS formation through the Fenton reaction. Additionally, cisplatin upregulates specific ferroptosis-related genes and pathways, such as ACSL4, which facilitates the incorporation of PUFAs into phospholipids, making them more susceptible to peroxidation (Ref. 99). Cisplatin can also induce the formation of inflammasomes, such as the NLRP3 inflammasome, which leads to the activation of caspase-1. Activated caspase-1 processes pro-inflammatory cytokines IL-1β and IL-18 and cleaves GSDMD, a key executor of pyroptosis (Refs. 100–102).
3. Arsenic trioxide (ATO): ATO has proven remarkably effective in treating acute promyelocytic leukaemia (APL), a distinct subset of acute myeloid leukaemia (AML), which makes up approximately 10% of AML cases in the US and accounts for about 1.2% of cancer-related deaths. ATO has been successful in inducing complete remission in APL patients, showing minimal myelosuppression and relatively mild side effects. These outcomes have cemented ATO as a potent monotherapy option for APL, especially as it promotes remission by degrading the PML-retinoic acid receptor alpha (PML-RARα) fusion protein, a critical driver of APL pathology. The promising results in APL have led to deeper exploration into ATO’s mechanisms, revealing broad effects on multiple cellular pathways involved in apoptosis, cell growth, angiogenesis and differentiation (Ref. 103). In leukaemia models like HL-60 cells, ATO has been shown to induce apoptosis through oxidative stress and the intrinsic mitochondrial pathway (Ref. 104). ATO’s potential has also extended to solid tumours, such as HCC and colon cancer. In HCC, ATO’s cytotoxicity is linked to oxidative stress, with increased levels of MDA, reduced antioxidant enzyme activities and elevated apoptotic markers like caspase 3 and TP53, suggesting that oxidative stress plays a key role in its cytotoxic mechanism (Ref. 105). In colon cancer cell models, ATO showed growth inhibition in a concentration-dependent manner, particularly targeting glutathione-deficient cells through ROS accumulation and caspase activation, underscoring its ability to leverage oxidative vulnerabilities in certain cancer cells (Ref. 106). ATO has also emerged as a potent anti-cancer agent capable of activating multiple non-apoptotic cell death pathways, with a particular emphasis on ferroptosis across various cancer types, including neuroblastoma (NB) (Refs. 107, 108), lung adenocarcinoma stem cells (LASCs) (Ref. 109) and HCC (Ref. 110). Beyond ferroptosis, ATO was shown to activate additional non-apoptotic pathways, including necroptosis and autophagy in certain cancers. This multi-pathway cytotoxicity enhances the immunogenicity of cancer cells, triggering the release of ‘danger signals’ that boost the immune response (Ref. 111). Thomas-Schoemann et al. have examined ATO’s immunomodulatory properties, showing it can reduce regulatory T cells (T_regs) and bolster antitumour immune responses. This immune enhancement presents opportunities for ATO’s integration with immunotherapies, potentially expanding its effectiveness in solid tumours (Ref. 112). Preclinical studies suggest that ATO when combined with immunotherapeutic agents like PD-1 inhibitors, can enhance immunosurveillance by fostering the recruitment of CD8+ T cells and activating interferon signalling pathways. For instance, ATO preconditioning has been shown to support the generation of whole-cell tumour vaccines, which can heighten immune cell recognition and engagement with cancer cells, potentially enhancing overall treatment efficacy (Ref. 111). In HCC models, the deployment of nanoparticle-based delivery systems for ATO has been shown to improve its tumour-specific targeting while minimizing systemic toxicity. These delivery systems augment ferroptosis induction in the tumour microenvironment, capitalizing on ATO’s ROS-driven mechanisms while reducing collateral damage to healthy cells. This selective ferroptosis induction by ATO, coupled with its immune-stimulatory effects, provides a versatile approach for overcoming apoptosis resistance in cancer cells. ATO has recently been also identified as an inducer of pyroptosis, as evidenced by cleavage of GSDME by caspase-3 in treated astroglioma cells (U251) and their rupture in response to the treatment. This dual role of caspase-3 highlights the flexibility of ATO’s mechanisms, enabling it to activate both apoptotic and pyroptotic pathways depending on the cellular context. Importantly, pyroptosis releases inflammatory mediators that can stimulate immune cell recruitment, potentially enhancing anti-tumour immunity by making the tumour microenvironment more immunogenic. This inflammatory response could attract immune cells to target the tumour more effectively (Ref. 113).

These findings indicate that oxidative stress is a key factor in how chemotherapeutic drugs work, acting as a central trigger for different types of cell death regardless of their primary mechanisms. Many of these drugs also have hemotoxic effects, largely due to increased ROS levels and weakened antioxidant defences. This imbalance can damage blood cells, leading to side effects such as anaemia, leukopenia and thrombocytopenia (Ref. 14). Understanding the exact mechanisms and pathways in which ROS are involved, including drug-induced oxeiptosis could provide valuable insights for enhancing cancer treatment strategies and minimizing side effects.

During chemotherapy, oxidative stress generates electrophilic aldehydes via lipid peroxidation, which damage cellular components and contribute to toxicity. Additionally, oxidative stress-derived substances can interfere with the progression of the cell cycle in cancer cells, often causing cell cycle checkpoint arrest. However, these aldehydes may also impair drug-induced apoptosis by deactivating death receptors and inhibiting caspase activation, which can ultimately reduce the effectiveness of the treatment. The activation of alternative cell death pathways, such as oxeiptosis, may offer a potential strategy to overcome resistance to treatment and improve therapeutic outcomes (Ref. 114).

The limited effectiveness of pro-oxidant therapies is a growing concern, as some cancer cells adapt and become insensitive to oxidative stress. Although ROS induce stress, they also promote resistance by activating antioxidant pathways or enabling phenotypic plasticity. Cancer stem cells (CSCs) are highly adaptable, self-renewing cells that drive tumour growth and therapy resistance. Their resilience stems from enhanced DNA repair capacity and efficient ROS scavenging, allowing them to survive oxidative stress. ROS can also promote CSCs formation and epithelial-mesenchymal transition (EMT), increasing resistance to treatment. Enhanced antioxidant capacity and quiescence further help CSCs evade damage from pro-oxidant therapies. As a result, targeting these protective mechanisms with more precise strategies is essential to improve treatment effectiveness. Additionally, recent research suggests that prolonged chemotherapy can lower the total ROS levels in cancer cells. These reduced ROS levels are thought to contribute to the development of drug resistance during chemotherapy, a topic that has been extensively discussed in recent literature (Refs. 14, 115–118). Contrary to the basic use of pro-oxidants and anti-oxidants, certain redox-active drugs can operate as adjuvants in cancer treatment. They can safeguard healthy tissues from oxidative stress damage, while also maintaining or improving the effectiveness of tumour radiation and chemotherapy, as reviewed by Jiang et al. (Ref. 14).

Recently, there have been reports of new medications and repurposed drugs that have been shown to have excellent pro-oxidant qualities and produce impressive therapeutic results. Elesclomol (STA-4783) is a copper ionophore that has been shown to enhance the therapeutic efficacy of PTX in patients with solid tumours (Ref. 119) and melanoma (Refs. 120, 121). This is achieved by inducing oxidative stress (Refs. 122, 123), mitochondrial dysfunction (Ref. 124), apoptosis (Ref. 122) and cuproptosis (Refs. 125, 126). Gao et al. discovered that elesclomol triggers ferroptosis in colorectal cancer cells via a copper-dependent mechanism. This is accomplished by promoting the breakdown of Cu-transporting ATPase 1, which then results in the production of ROS (Ref. 127). Elesclomol is the most advanced Cu ionophore currently being studied for its ability to work synergistically with paclitaxel and carboplatin in a clinical setting. Currently, there are nine registered clinical trials on ClinicalTrials.gov website (https://clinicaltrials.gov/search?cond=cancer&intr=Elesclomol) that aim to use elesclomol for cancer treatment. Among them, six were completed, two were suspended/terminated, and one had an unknown status (accessed on 23.01.2026). These trials were largely mentioned in the earlier reviews conducted by Zheng et al. (Ref. 128), Tarin et al. (Ref. 129) or our group (Ref. 12), and the results are summarized in Table 2.

**Table 2.** Summary of clinical studies exploring elesclomol for cancer treatment

Tested agents	PMID/NCT	Phase	Population/Tumour Type	Outcome/Notes
Elesclomol + paclitaxel	PMID:17255281 NCT00088114	I	Adults with refractory solid tumours	Maximal tolerated dose of elesclomol = 438 mg/m^2^; toxicities typical of paclitaxel; partial responses in Kaposi’s sarcoma and ovarian cancer; elesclomol had linear pharmacokinetics; paclitaxel clearance decreased with elesclomol escalation
Elesclomol + paclitaxel	PMID:19826135 NCT00522834	II	Stage IV metastatic melanoma	Median progression-free survival of 112 vs 56 days; response rate 15% vs 3%; median overall survival of 11.9 vs 7.8 months; well-tolerated, encouraging result prompting phase III clinical trials.
Elesclomol + paclitaxel)	PMID:23401447 NCT00522834	III	Chemotherapy-naive advanced melanoma	Did not meet the primary progression-free survival endpoint; terminated early due to overall survival imbalance favouring paclitaxel, especially in the high lactate dehydrogenase subgroup; the normal lactate dehydrogenase subgroup showed a progression-free survival benefit.
Elesclomol + paclitaxel	PMID:30309721 NCT00888615	II	Platinum-resistant recurrent ovarian/tubal/peritoneal cancer	Overall response rate of 19.6%, median progression-free survival of 3.6 months; median overall survival of 13.3 months; combination well tolerated but showed insufficient activity to justify further study.

Disulfiram (DSF), traditionally used in the treatment of chronic alcoholism, has emerged as a promising anticancer agent when combined with copper ions. This combination forms a copper diethyldithiocarbamate complex, Cu(DDC)₂ or CuET, which demonstrates potent anticancer properties by inhibiting NF-κB, the ubiquitin-proteasome system and modulating ROS levels. These actions disrupt several molecular pathways involved in drug resistance, stemness, angiogenesis and metastasis. Various clinical trials have investigated the efficacy of DSF/Cu across a range of cancer types, including metastatic castrate-resistant prostate cancer (CRPC), metastatic breast cancer and glioblastoma. While preclinical studies have shown promising results, some clinical trials have been terminated due to a lack of efficacy or other issues, highlighting the challenges of translating these findings into clinical success. One major obstacle has been the instability and rapid metabolism of DSF, which has prompted the development of novel formulations, such as liposomal encapsulation, to enhance its delivery and effectiveness. The strategic repurposing of DSF, in combination with copper supplementation, takes advantage of existing safety profiles and pharmacokinetic data, offering a cost-effective and potentially faster path to developing new cancer therapies. These therapies could induce ferroptosis and cuproptosis, providing alternative mechanisms to overcome resistance to conventional treatments. This topic was recently reviewed by our group (Ref. 12) or other authors (Refs. 130, 131) and is provided in Table 3.

**Table 3.** Summary of clinical studies exploring disulfiram for cancer treatment

Tested agents	PMID/NCT	Phase	Population/Tumour Type	Outcome/Notes
Disulfiram + cisplatin	PMID: 2180271	Randomized prospective	Cisplatin-sensitive malignancies	No improvement in response, progression-free survival, or overall survival. No nephroprotection. Increased gastrointestinal toxicity and ototoxicity with disulfiram.
Disulfiram monotherapy	PMID: 23958896 NCT01118741	Phase I (dose escalation)	Non-metastatic recurrent prostate cancer	Minor, transient peripheral blood mononuclear cell demethylation in a minority of patients. No prostate-specific antigen response or clinical benefit. Poor tolerability; further development not recommended.
Disulfiram + cisplatin + vinorelbine	PMID: 25777347 NCT00312819	Phase II	Newly diagnosed advanced non–small cell lung cancer	Well tolerated. Improved median overall survival (10 vs 7.1 months). Two long-term survivors in the disulfiram treatment arm; small study.
Disulfiram + temozolomide	PMID: 26966095	Phase I	Newly diagnosed glioblastoma multiforme (post-chemoradiotherapy)	Maximum tolerated dose = 500 mg/day. Reversible neurologic toxicities. Limited proteasome inhibition; modest progression-free survival.
Disulfiram ± copper + temozolomide	PMID: 29374809 NCT03034135	Phase I (dose escalation + expansion)	Newly diagnosed glioblastoma multiforme	500 mg/day tolerated; 1000 mg/day not. Copper did not enhance efficacy or toxicity. Outcomes similar to historical controls.
Disulfiram + copper + temozolomide	PMID: 30771200 NCT03034135	Phase II	Recurrent temozolomide-resistant glioblastoma multiforme	Overall response rate of 0%, clinical benefit of 14%, median progression-free survival of 1.7 months, median overall survival of 7.1 months. Well tolerated but showed limited activity.
Disulfiram + copper gluconate	PMID: 33957901 NCT00742911	Phase I	Advanced solid tumours with liver involvement	Well tolerated up to Cu 8 mg. No objective responses; some stable disease. Increased protein S-glutathionylation.
Disulfiram + copper	PMID: 35286730	Phase Ib	Metastatic castration-resistant prostate cancer	No positive prostate-specific antigen or radiographic responses. Rapid disulfiram metabolism to inactive methyl diethyldithiocarbamate. Lack of efficacy.
Disulfiram + cisplatin	PMID: 35763178 NCT03950830	Phase II	Relapsed/refractory germ cell tumours	No objective responses; trial stopped early. Limited disease stabilization; manageable toxicity.
Disulfiram + copper + alkylating chemotherapy	PMID: 37000452 NCT02678975	Phase II/III (randomized)	Recurrent glioblastoma multiforme	No survival benefit. Higher grade ≥ 3 and serious adverse effects. Increased treatment discontinuation.
Disulfiram + copper + RT + temozolomide	PMID: 38768767	Phase I/II	Newly diagnosed glioblastoma multiforme	Maximum tolerated dose = 375 mg/day. Limited efficacy overall. Prolonged remission in B-Raf proto-oncogene, serine/threonine kinase (BRAF)-mutant glioblastoma multiforme subgroup.

**Figure 3.**
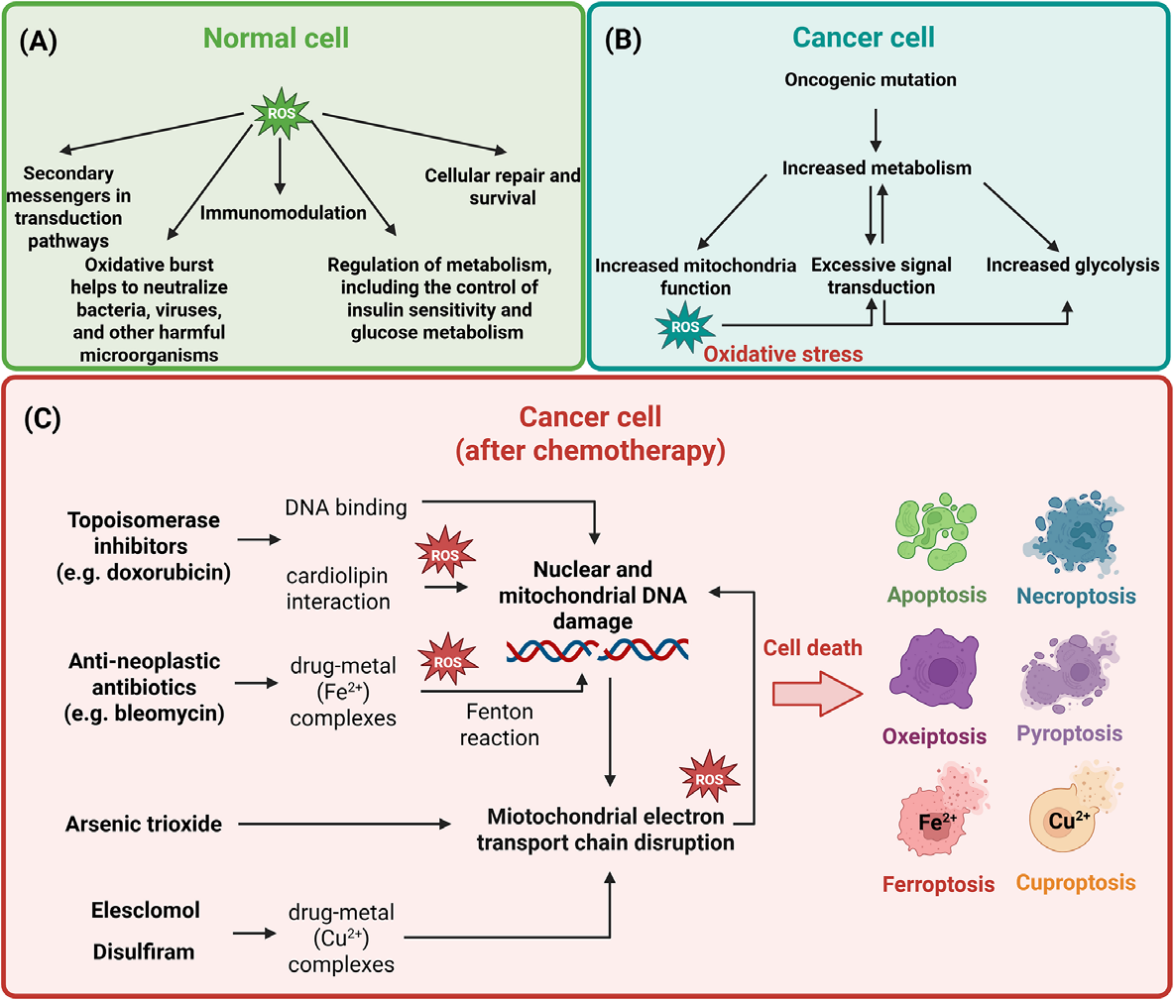
Reactive oxygen species (ROS) and their role in normal (A) and cancer cells before (B) and after (C) introduction of chemotherapeutical agents. In normal cells, ROS levels are tightly controlled, supporting physiological processes such as signal transduction, metabolic regulation, immune defence, cellular repair and survival. In cancer cells, oncogenic mutations, increased mitochondrial activity, glycolytic shifts and dysregulated signalling pathways drive excessive ROS production, which leads to oxidative stress. Chemotherapeutic agents exploit this vulnerability by further increasing ROS to lethal levels. Topoisomerase inhibitors (e.g. doxorubicin) generate ROS via redox cycling, causing DNA damage and triggering cell death; anti-neoplastic antibiotics (e.g. bleomycin) induce ROS through metal ion chelation; arsenic trioxide disrupts mitochondrial function, contributing to the elevated ROS production; elesclomol and disulfiram form complexes with Cu^2+^, enhancing mitochondrial ROS production. The resulting ROS-mediated cell deaths include apoptosis, necroptosis, ferroptosis, cuproptosis, pyroptosis and potentially oxeiptosis. Inspired by (Ref. 14). Created in BioRender. Kciuk, M. (2025) https://BioRender.com/ppoudr8.

Agents such as elesclomol and DSF in combination with copper exhibit pronounced pro-oxidant properties, primarily through the induction of intracellular ROS, mitochondrial dysfunction and redox imbalance, hallmark triggers of oxeiptosis. The mechanisms of action of different classes of chemotherapeutical drugs involving oxidative stress induction that lead to various types of cell death are shown in Figure 3.

The NeoSAC trial introduced a novel perspective on the role of oxeiptosis in cancer treatment in response to neoadjuvant therapy for triple-negative breast cancer (TNBC). Oxeiptosis appeared to correlate with treatment response, as patients with higher baseline oxeiptosis scores were more likely to achieve a pathological complete response (pCR). Interestingly, post-treatment analysis revealed a decrease in oxeiptosis scores in the pCR group, while the non-pCR group exhibited an increase, suggesting a dynamic relationship between tumour cell susceptibility to oxidative stress and treatment efficacy. While the mechanistic link between oxeiptosis and antiangiogenic or immune checkpoint inhibitor therapy remains largely unexplored, the study’s findings suggest a potential interplay between oxidative stress modulation and immune activation. Prior research indicates that alloimperatorin can induce oxeiptosis in breast cancer cells, restraining their proliferation and invasion. Furthermore, KEAP1 may play a role in this process, although its direct association with oxeiptosis remains to be fully elucidated. These findings open new avenues for investigation, particularly in assessing whether modulating oxeiptosis can enhance responses to immunotherapy and antiangiogenic treatment. While the current observations are derived from a TNBC-specific neoadjuvant setting, TNBC represents a biologically aggressive and immunologically active tumour type, making it a suitable model for exploring stress-related cell death pathways in the context of combination therapies. Importantly, oxeiptosis is not unique to TNBC, and similar mechanisms of oxidative stress regulation and immune modulation may be operative in other malignancies with high unmet clinical needs, such as pancreatic cancer and glioblastoma. Future research should therefore focus on elucidating the molecular mechanisms governing oxeiptosis, validating its predictive value across tumour types and determining its potential as a therapeutic target. A deeper understanding of how oxeiptosis influences the tumour microenvironment may ultimately contribute to the development of more effective, biomarker-driven treatment strategies across multiple cancers (Ref. 132).

## Challenges, prospects and summary

While chemotherapeutic drugs are known for their ability to induce oxidative stress, further research is necessary to determine whether they specifically trigger oxeiptosis, as opposed to other forms of cell death, such as apoptosis, necroptosis, pyroptosis, ferroptosis or cuproptosis. Inducing oxeiptosis in cancer cells offers several potential advantages:

1. Oxeiptosis has been proposed to preferentially affect cells exposed to excessive oxidative stress; however, tumour cells can variably adapt by enhancing antioxidant defences, which may limit their susceptibility to this form of cell death. In contrast, normal cells generally maintain tighter redox homeostasis, potentially reducing unintended cytotoxicity. Thus, the extent to which oxeiptosis selectively targets cancer cells likely depends on the balance between oxidative stress burden and the capacity for antioxidant adaptation.
2. Unlike necrosis, which often results in significant inflammation, oxeiptosis is a controlled process that minimizes inflammatory responses.
3. ROS are involved in cellular signalling and have been implicated in cancer cell proliferation, metastasis and genomic instability. Although oxeiptosis represents a regulated response to elevated oxidative stress, its impact on tumour progression is currently speculative and requires further experimental validation.
4. Drugs that induce oxeiptosis could be used in combination with other treatments (e.g. chemotherapy, radiotherapy), enhancing overall efficacy and potentially overcoming resistance mechanisms.

However, challenges remain, such as the potential damage to normal cells, the development of oxeiptosis resistance mechanisms, and the incomplete understanding of the process. These hurdles must be addressed before oxeiptosis-inducing agents can be introduced into clinical practice. To date, no clinical trials (besides NeoSAC) specifically targeting the induction of oxeiptosis in cancer cells have been conducted. Oxeiptosis remains a relatively new field of research, still in the preclinical stage. While it holds promise as a mechanism for inducing cell death in cancer cells, the focus remains on understanding the underlying mechanisms and identifying potential therapeutic triggers.

Given the role of ROS in inducing oxeiptosis, there is considerable potential for drugs that modulate ROS levels to be investigated for their ability to trigger this form of cell death in cancer cells. Despite its infancy, oxeiptosis is garnering growing scientific interest. As of now (23.01.2026), there are 80 works mentioning the term ‘oxeiptosis’ in the PubMed database (pubmed.ncbi.nlm.nih.gov). This figure reflects both the increasing attention to the concept and the potential for future developments in this area.

There are several directions to explore, not only to deepen our understanding of the process but also to apply this knowledge to cancer treatment (Refs. 46, 133):

1. Different cancers exhibit unique patterns of ROS generation and antioxidant capacities. Therefore, targeting with agents based on these cancer-specific ROS signatures could enhance treatment selectivity. Comprehensive profiling of ROS-related biomarkers by cancer type would help identify patients most likely to benefit from ROS-targeted therapies.
2. Efforts should focus on designing advanced drug delivery methods, such as nanotechnology-based systems or antibody-drug conjugates, to specifically target ROS in tumour cells. For instance, nanoparticles engineered to respond to specific ROS thresholds in cancer cells could release therapeutic agents only when these conditions are met, improving efficacy and reducing off-target effects across different cancer types.
3. Cancer cells often upregulate specific antioxidant enzymes, such as GPX and SOD, to cope with elevated ROS levels. Developing inhibitors or modulators that target these enzymes could selectively impair the survival of cancer cells without damaging normal cells with lower (normal/baseline) ROS levels.
4. The adaptive regulation of the NRF2 pathway plays a role in cancer cell resistance to oxidative stress. Small-molecule inhibitors that transiently suppress NRF2 activity could enhance ROS accumulation selectively in cancer cells, making them more susceptible to oxidative damage.
5. Combining ROS-generating agents with inhibitors of antioxidant pathways that are preferentially utilized by certain cancer subtypes could constitute a synthetic lethal strategy. However, the identity and therapeutic relevance of these dependencies are still unknown and may vary across tumours, underscoring the need for subtype-specific functional studies.

## Abbreviations

4-HNE: 4-hydroxynonenal
5FU: 5-fluorouracil
5FU-CS-MNPs: 5-fluorouracil-chitosan-coated magnetic nanoparticles complex
ACSL4: long-chain acyl-CoA synthetase family 4
AIFM1: mitochondrial apoptosis-inducing factor 1
ALDH: aldehyde dehydrogenase
ALT: alantolactone
AML: acute myeloid leukaemia
AREs: antioxidant response elements
AIM2: absent in melanoma 2
AP-1: activator protein-1
ATO: arsenic trioxide
BAK1: Bcl-2 homologous antagonist/killer 1
BPH: benign prostatic hyperplasia
BRAF: B-Raf proto-oncogene, serine/threonine kinase
C-PARP: cleaved poly(ADP-ribose) polymerase
CRC: colorectal cancer
CRPC: castrate-resistant prostate cancer
CSCs: cancer stem cells
CS-MNPs: chitosan-coated magnetic nanoparticles
Cu(DDC)₂: copper diethyldithiocarbamate
CuET: copper diethyldithiocarbamate complex
DAMPs: danger-associated molecular patterns
DFNA5: deafness autosomal dominant 5
DOX: doxorubicin
DSF: disulfiram
EMT: epithelial-mesenchymal transition
ER: endoplasmic reticulum
ETC: electron transport chain
FER-1: ferrostatin-1
FSP1: ferroptosis suppressor protein 1
GSH: glutathione
GSDMD: gasdermin D
GPX: glutathione peroxidase
GSS: glutathione synthetase
GST: glutathione S-transferase
γ-GCS: gamma-glutamylcysteine synthetase
HCC: hepatocellular carcinoma
HSP70/90: heat shock protein 70/90
ICG: indocyanine green
IL-1β: interleukin 1 beta
IL-18: interleukin 18
IRP1: iron regulatory protein 1
IRP2: iron regulatory protein 2
JNK: Jun N-terminal kinase
KEAP1: Kelch-like ECH-associated protein 1
LASCs: lung adenocarcinoma stem cells
LPCAT3: lysophosphatidylcholine acyltransferase 3
MAPK: mitogen-activated protein kinases
MDA: malondialdehyde
MLKL: mixed lineage kinase domain-like protein
MOMP: mitochondrial outer membrane permeabilization
MPTP: mitochondrial permeability transition pore
MTD: maximum tolerated dose
M-ICG-PTX: modified lipid nanoparticles encapsulating indocyanine green and paclitaxel
mtDNA: mitochondrial DNA
NB: neuroblastoma
NAC: N-acetylcysteine
NF-κB: nuclear factor kappa-light-chain-enhancer of activated B cells
NLRP3: NOD-, LRR- and pyrin domain-containing protein 3/nucleotide-binding domain, leucine-rich–containing family, pyrin domain–containing-3/ nucleotide-binding, oligomerization domain-like receptor family pyrin domain containing 3
nDNA: nuclear DNA
NOX: NADPH oxidase
NRF2: nuclear factor erythroid 2-related factor 2
OTUD1: OTU deubiquitinase 1
PAMPs: pathogen-associated molecular patterns
PARP: poly-ADP ribose polymerase
pCR: pathological complete response
PEITC: phenethyl isothiocyanate
PGAM5: phosphoglycerate mutase family member 5
PRX: peroxiredoxin
PTT: photothermal therapy
PTX: paclitaxel
PUFAs: polyunsaturated fatty acids
PUMA: p53 upregulated modulator of apoptosis
P-gp: P-glycoprotein
ROS: reactive oxygen species
RIPKs: receptor-interacting protein kinases
SDT: sonodynamic therapy
SLC7A11: solute carrier family 7, subfamily A, member 11
SNG: sanguinarine
SOD: superoxide dismutase
STA-4783: elesclomol
TNBC: triple-negative breast cancer
TP53: cellular tumour antigen p53
TPP: triphenylphosphine
T_regs: regulatory T cells
TKIs: tyrosine kinase inhibitors
TNF: tumour necrosis factor
UPR: unfolded protein response
Z-VAD-FMK: apoptosis inhibitor (N-benzyloxycarbonyl-Val-Ala-Asp-fluoromethyl ketone).
